# Publisher Correction: Genomic reconstruction of the SARS CoV-2 epidemic in England

**DOI:** 10.1038/s41586-022-04887-8

**Published:** 2022-06-07

**Authors:** Harald S. Vöhringer, Theo Sanderson, Matthew Sinnott, Nicola De Maio, Thuy Nguyen, Richard Goater, Frank Schwach, Ian Harrison, Joel Hellewell, Cristina V. Ariani, Sonia Gonçalves, David K. Jackson, Ian Johnston, Alexander W. Jung, Callum Saint, John Sillitoe, Maria Suciu, Nick Goldman, Jasmina Panovska-Griffiths, The Wellcome Sanger Institute COVID-19 Surveillance Team, Irina Abnizova, Louise Aigrain, Alex Alderton, Mozam Ali, Laura Allen, Roberto Amato, Ralph Anderson, Cristina Ariani, Siobhan Austin-Guest, Sendu Bala, Jeffrey Barrett, Andrew Bassett, Kristina Battleday, James Beal, Mathew Beale, Charlotte Beaver, Sam Bellany, Tristram Bellerby, Katie Bellis, Duncan Berger, Matt Berriman, Emma Betteridge, Paul Bevan, Simon Binley, Jason Bishop, Kirsty Blackburn, James Bonfield, Nick Boughton, Sam Bowker, Timothy Brendler-Spaeth, Iraad Bronner, Tanya Brooklyn, Sarah Kay Buddenborg, Robert Bush, Catarina Caetano, Alex Cagan, Nicola Carter, Joanna Cartwright, Tiago Carvalho Monteiro, Liz Chapman, Tracey-Jane Chillingworth, Peter Clapham, Richard Clark, Adrian Clarke, Catriona Clarke, Daryl Cole, Elizabeth Cook, Maria Coppola, Linda Cornell, Clare Cornwell, Craig Corton, Abby Crackett, Alison Cranage, Harriet Craven, Sarah Craw, Mark Crawford, Tim Cutts, Monika Dabrowska, Matt Davies, Robert Davies, Joseph Dawson, Callum Day, Aiden Densem, Thomas Dibling, Cat Dockree, David Dodd, Sunil Dogga, Matthew Dorman, Gordon Dougan, Martin Dougherty, Alexander Dove, Lucy Drummond, Eleanor Drury, Monika Dudek, Jillian Durham, Laura Durrant, Elizabeth Easthope, Sabine Eckert, Pete Ellis, Ben Farr, Michael Fenton, Marcella Ferrero, Neil Flack, Howerd Fordham, Grace Forsythe, Luke Foulser, Matt Francis, Audrey Fraser, Adam Freeman, Anastasia Galvin, Maria Garcia-Casado, Alex Gedny, Sophia Girgis, James Glover, Sonia Goncalves, Scott Goodwin, Oliver Gould, Marina Gourtovaia, Andy Gray, Emma Gray, Coline Griffiths, Yong Gu, Florence Guerin, Will Hamilton, Hannah Hanks, Ewan Harrison, Alexandria Harrott, Edward Harry, Julia Harvison, Paul Heath, Anastasia Hernandez-Koutoucheva, Rhiannon Hobbs, Dave Holland, Sarah Holmes, Gary Hornett, Nicholas Hough, Liz Huckle, Lena Hughes-Hallet, Adam Hunter, Stephen Inglis, Sameena Iqbal, Adam Jackson, David Jackson, Keith James, Dorota Jamrozy, Carlos Jimenez Verdejo, Matthew Jones, Kalyan Kallepally, Leanne Kane, Keely Kay, Sally Kay, Jon Keatley, Alan Keith, Alison King, Lucy Kitchin, Matt Kleanthous, Martina Klimekova, Petra Korlevic, Ksenia Krasheninnkova, Greg Lane, Cordelia Langford, Adam Laverack, Katharine Law, Mara Lawniczak, Stefanie Lensing, Steven Leonard, Laura Letchford, Kevin Lewis, Amanah Lewis-Wade, Jennifer Liddle, Quan Lin, Sarah Lindsay, Sally Linsdell, Rich Livett, Stephanie Lo, Rhona Long, Jamie Lovell, Jon Lovell, Catherine Ludden, James Mack, Mark Maddison, Aleksei Makunin, Irfan Mamun, Jenny Mansfield, Neil Marriott, Matt Martin, Matthew Mayho, Shane McCarthy, Jo McClintock, Samantha McGuigan, Sandra McHugh, Liz McMinn, Carl Meadows, Emily Mobley, Robin Moll, Maria Morra, Leanne Morrow, Kathryn Murie, Sian Nash, Claire Nathwani, Plamena Naydenova, Alexandra Neaverson, Rachel Nelson, Ed Nerou, Jon Nicholson, Tabea Nimz, Guillaume G. Noell, Sarah O’Meara, Valeriu Ohan, Karen Oliver, Charles Olney, Doug Ormond, Agnes Oszlanczi, Steve Palmer, Yoke Fei Pang, Barbora Pardubska, Naomi Park, Aaron Parmar, Gaurang Patel, Minal Patel, Maggie Payne, Sharon Peacock, Arabella Petersen, Deborah Plowman, Tom Preston, Liam Prestwood, Christoph Puethe, Michael Quail, Diana Rajan, Shavanthi Rajatileka, Richard Rance, Suzannah Rawlings, Nicholas Redshaw, Joe Reynolds, Mark Reynolds, Simon Rice, Matt Richardson, Connor Roberts, Katrina Robinson, Melanie Robinson, David Robinson, Hazel Rogers, Eduardo Martin Rojo, Daljit Roopra, Mark Rose, Luke Rudd, Ramin Sadri, Nicholas Salmon, David Saul, Frank Schwach, Carol Scott, Phil Seekings, Lesley Shirley, Alison Simms, Matthew Sinnott, Shanthi Sivadasan, Bart Siwek, Dale Sizer, Kenneth Skeldon, Jason Skelton, Joanna Slater-Tunstill, Lisa Sloper, Nathalie Smerdon, Chris Smith, Christen Smith, James Smith, Katie Smith, Michelle Smith, Sean Smith, Tina Smith, Leighton Sneade, Carmen Diaz Soria, Catarina Sousa, Emily Souster, Andrew Sparkes, Michael Spencer-Chapman, Janet Squares, Robert Stanley, Claire Steed, Tim Stickland, Ian Still, Michael R. Stratton, Michelle Strickland, Allen Swann, Agnieszka Swiatkowska, Neil Sycamore, Emma Swift, Edward Symons, Suzanne Szluha, Emma Taluy, Nunu Tao, Katy Taylor, Sam Taylor, Stacey Thompson, Mark Thompson, Mark Thomson, Nicholas Thomson, Scott Thurston, Gerry Tonkin-Hill, Dee Toombs, Benjamin Topping, Jaime Tovar-Corona, Daniel Ungureanu, James Uphill, Jana Urbanova, Philip Jansen Van Vuuren, Valerie Vancollie, Paul Voak, Danielle Walker, Matthew Walker, Matt Waller, Gary Ward, Charlie Weatherhogg, Niki Webb, Danni Weldon, Alan Wells, Eloise Wells, Luke Westwood, Theo Whipp, Thomas Whiteley, Georgia Whitton, Andrew Whitwham, Sara Widaa, Mia Williams, Mark Wilson, Sean Wright, The COVID-19 Genomics UK (COG-UK) Consortium*, Samuel C. Robson, Thomas R. Connor, Nicholas J. Loman, Tanya Golubchik, Rocio T. Martinez Nunez, David Bonsall, Andrew Rambaut, Luke B. Snell, Catherine Ludden, Sally Corden, Eleni Nastouli, Gaia Nebbia, Katrina Lythgoe, M. Estee Torok, Ian G. Goodfellow, Jacqui A. Prieto, Kordo Saeed, Catherine Houlihan, Dan Frampton, William L. Hamilton, Adam A. Witney, Giselda Bucca, Cassie F. Pope, Catherine Moore, Emma C. Thomson, Ewan M. Harrison, Colin P. Smith, Fiona Rogan, Shaun M. Beckwith, Abigail Murray, Dawn Singleton, Kirstine Eastick, Liz A. Sheridan, Paul Randell, Leigh M. Jackson, Sónia Gonçalves, Derek J. Fairley, Matthew W. Loose, Joanne Watkins, Samuel Moses, Sam Nicholls, Matthew Bull, Darren L. Smith, David M. Aanensen, Dinesh Aggarwal, James G. Shepherd, Martin D. Curran, Surendra Parmar, Matthew D. Parker, Catryn Williams, Sharon Glaysher, Anthony P. Underwood, Matthew Bashton, Nicole Pacchiarini, Katie F. Loveson, Matthew Byott, Alessandro M. Carabelli, Kate E. Templeton, Thushan I. de Silva, Dennis Wang, Cordelia F. Langford, Rory N. Gunson, Simon Cottrell, Justin O’Grady, Dominic Kwiatkowski, Patrick J. Lillie, Nicholas Cortes, Nathan Moore, Claire Thomas, Phillipa J. Burns, Tabitha W. Mahungu, Steven Liggett, Angela H. Beckett, Matthew T. G. Holden, Lisa J. Levett, Husam Osman, Mohammed O. Hassan-Ibrahim, David A. Simpson, Meera Chand, Ravi K. Gupta, Alistair C. Darby, Steve Paterson, Oliver G. Pybus, Erik Volz, Daniela de Angelis, David L. Robertson, Andrew J. Page, Andrew R. Bassett, Nick Wong, Yusri Taha, Michelle J. Erkiert, Michael H. Spencer Chapman, Rebecca Dewar, Martin P. McHugh, Siddharth Mookerjee, Stephen Aplin, Matthew Harvey, Thea Sass, Helen Umpleby, Helen Wheeler, James P. McKenna, Ben Warne, Joshua F. Taylor, Yasmin Chaudhry, Rhys Izuagbe, Aminu S. Jahun, Gregory R. Young, Claire McMurray, Clare M. McCann, Andrew Nelson, Scott Elliott, Hannah Lowe, Anna Price, Matthew R. Crown, Sara Rey, Sunando Roy, Ben Temperton, Sharif Shaaban, Andrew R. Hesketh, Kenneth G. Laing, Irene M. Monahan, Judith Heaney, Emanuela Pelosi, Siona Silviera, Eleri Wilson-Davies, Helen Fryer, Helen Adams, Louis du Plessis, Rob Johnson, William T. Harvey, Joseph Hughes, Richard J. Orton, Lewis G. Spurgin, Yann Bourgeois, Chris Ruis, Áine O’Toole, Theo Sanderson, Christophe Fraser, Jonathan Edgeworth, Judith Breuer, Stephen L. Michell, John A. Todd, Michaela John, David Buck, Kavitha Gajee, Gemma L. Kay, Sharon J. Peacock, David Heyburn, Katie Kitchman, Alan McNally, David T. Pritchard, Samir Dervisevic, Peter Muir, Esther Robinson, Barry B. Vipond, Newara A. Ramadan, Christopher Jeanes, Jana Catalan, Neil Jones, Ana da Silva Filipe, Chris Williams, Marc Fuchs, Julia Miskelly, Aaron R. Jeffries, Naomi R. Park, Amy Ash, Cherian Koshy, Magdalena Barrow, Sarah L. Buchan, Anna Mantzouratou, Gemma Clark, Christopher W. Holmes, Sharon Campbell, Thomas Davis, Ngee Keong Tan, Julianne R. Brown, Kathryn A. Harris, Stephen P. Kidd, Paul R. Grant, Li Xu-McCrae, Alison Cox, Pinglawathee Madona, Marcus Pond, Paul A. Randell, Karen T. Withell, Cheryl Williams, Clive Graham, Rebecca Denton-Smith, Emma Swindells, Robyn Turnbull, Tim J. Sloan, Andrew Bosworth, Stephanie Hutchings, Hannah M. Pymont, Anna Casey, Liz Ratcliffe, Christopher R. Jones, Bridget A. Knight, Tanzina Haque, Jennifer Hart, Dianne Irish-Tavares, Eric Witele, Craig Mower, Louisa K. Watson, Jennifer Collins, Gary Eltringham, Dorian Crudgington, Ben Macklin, Miren Iturriza-Gomara, Anita O. Lucaci, Patrick C. McClure, Matthew Carlile, Nadine Holmes, Christopher Moore, Nathaniel Storey, Stefan Rooke, Gonzalo Yebra, Noel Craine, Malorie Perry, Nabil-Fareed Alikhan, Stephen Bridgett, Kate F. Cook, Christopher Fearn, Salman Goudarzi, Ronan A. Lyons, Thomas Williams, Sam T. Haldenby, Robert M. Davies, Rahul Batra, Beth Blane, Moira J. Spyer, Perminder Smith, Mehmet Yavus, Rachel J. Williams, Adhyana I. K. Mahanama, Buddhini Samaraweera, Sophia T. Girgis, Samantha E. Hansford, Angie Green, Katherine L. Bellis, Matthew J. Dorman, Joshua Quick, Radoslaw Poplawski, Nicola Reynolds, Andrew Mack, Arthur Morriss, Thomas Whalley, Bindi Patel, Iliana Georgana, Myra Hosmillo, Malte L. Pinckert, Joanne Stockton, John H. Henderson, Amy Hollis, William Stanley, Wen C. Yew, Richard Myers, Alicia Thornton, Alexander Adams, Tara Annett, Hibo Asad, Alec Birchley, Jason Coombes, Johnathan M. Evans, Laia Fina, Bree Gatica-Wilcox, Lauren Gilbert, Lee Graham, Jessica Hey, Ember Hilvers, Sophie Jones, Hannah Jones, Sara Kumziene-Summerhayes, Caoimhe McKerr, Jessica Powell, Georgia Pugh, Sarah Taylor, Alexander J. Trotter, Charlotte A. Williams, Leanne M. Kermack, Benjamin H. Foulkes, Marta Gallis, Hailey R. Hornsby, Stavroula F. Louka, Manoj Pohare, Paige Wolverson, Peijun Zhang, George MacIntyre-Cockett, Amy Trebes, Robin J. Moll, Lynne Ferguson, Emily J. Goldstein, Alasdair Maclean, Rachael Tomb, Igor Starinskij, Laura Thomson, Joel Southgate, Moritz U. G. Kraemer, Jayna Raghwani, Alex E. Zarebski, Olivia Boyd, Lily Geidelberg, Chris J. Illingworth, Chris Jackson, David Pascall, Sreenu Vattipally, Timothy M. Freeman, Sharon N. Hsu, Benjamin B. Lindsey, Jaime M. Tovar-Corona, MacGregor Cox, Khalil Abudahab, Mirko Menegazzo, Ben E. W. Taylor, Corin A. Yeats, Afrida Mukaddas, Derek W. Wright, Leonardo de Oliveira Martins, Rachel Colquhoun, Verity Hill, Ben Jackson, J. T. McCrone, Nathan Medd, Emily Scher, Jon-Paul Keatley, Tanya Curran, Sian Morgan, Patrick Maxwell, Ken Smith, Sahar Eldirdiri, Anita Kenyon, Alison H. Holmes, James R. Price, Tim Wyatt, Alison E. Mather, Timofey Skvortsov, John A. Hartley, Martyn Guest, Christine Kitchen, Ian Merrick, Robert Munn, Beatrice Bertolusso, Jessica Lynch, Gabrielle Vernet, Stuart Kirk, Elizabeth Wastnedge, Rachael Stanley, Giles Idle, Declan T. Bradley, Jennifer Poyner, Matilde Mori, Owen Jones, Victoria Wright, Ellena Brooks, Carol M. Churcher, Mireille Fragakis, Katerina Galai, Andrew Jermy, Sarah Judges, Georgina M. McManus, Kim S. Smith, Elaine Westwick, Stephen W. Attwood, Frances Bolt, Alisha Davies, Elen De Lacy, Fatima Downing, Sue Edwards, Lizzie Meadows, Sarah Jeremiah, Nikki Smith, Themoula Charalampous, Amita Patel, Louise Berry, Tim Boswell, Vicki M. Fleming, Hannah C. Howson-Wells, Amelia Joseph, Manjinder Khakh, Michelle M. Lister, Paul W. Bird, Karlie Fallon, Thomas Helmer, Claire L. McMurray, Mina Odedra, Jessica Shaw, Julian W. Tang, Nicholas J. Willford, Victoria Blakey, Veena Raviprakash, Nicola Sheriff, Lesley-Anne Williams, Theresa Feltwell, Luke Bedford, James S. Cargill, Warwick Hughes, Jonathan Moore, Susanne Stonehouse, Laura Atkinson, Jack C. D. Lee, Divya Shah, Adela Alcolea, Natasha Ohemeng-Kumi, John Ramble, Jasveen Sehmi, Rebecca Williams, Wendy Chatterton, Monika Pusok, William Everson, Anibolina Castigador, Emily Macnaughton, Kate El Bouzidi, Temi Lampejo, Malur Sudhanva, Cassie Breen, Graciela Sluga, Shazaad S. Y. Ahmad, Ryan P. George, Nicholas W. Machin, Debbie Binns, Victoria James, Rachel Blacow, Lindsay Coupland, Louise Smith, Edward Barton, Debra Padgett, Garren Scott, Aidan Cross, Mariyam Mirfenderesky, Jane Greenaway, Kevin Cole, Phillip Clarke, Nichola Duckworth, Sarah Walsh, Kelly Bicknell, Robert Impey, Sarah Wyllie, Richard Hopes, Chloe Bishop, Vicki Chalker, Laura Gifford, Zoltan Molnar, Cressida Auckland, Cariad Evans, Kate Johnson, David G. Partridge, Mohammad Raza, Paul Baker, Stephen Bonner, Sarah Essex, Leanne J. Murray, Andrew I. Lawton, Shirelle Burton-Fanning, Brendan A. I. Payne, Sheila Waugh, Andrea N. Gomes, Maimuna Kimuli, Darren R. Murray, Paula Ashfield, Donald Dobie, Fiona Ashford, Angus Best, Liam Crawford, Nicola Cumley, Megan Mayhew, Oliver Megram, Jeremy Mirza, Emma Moles-Garcia, Benita Percival, Leah Ensell, Helen L. Lowe, Laurentiu Maftei, Matteo Mondani, Nicola J. Chaloner, Benjamin J. Cogger, Lisa J. Easton, Hannah Huckson, Jonathan Lewis, Sarah Lowdon, Cassandra S. Malone, Florence Munemo, Manasa Mutingwende, Roberto Nicodemi, Olga Podplomyk, Thomas Somassa, Andrew Beggs, Alex Richter, Claire Cormie, Joana Dias, Sally Forrest, Ellen E. Higginson, Mailis Maes, Jamie Young, Rose K. Davidson, Kathryn A. Jackson, Lance Turtle, Alexander J. Keeley, Jonathan Ball, Timothy Byaruhanga, Joseph G. Chappell, Jayasree Dey, Jack D. Hill, Emily J. Park, Arezou Fanaie, Rachel A. Hilson, Geraldine Yaze, Safiah Afifi, Robert Beer, Joshua Maksimovic, Kathryn McCluggage Masters, Karla Spellman, Catherine Bresner, William Fuller, Angela Marchbank, Trudy Workman, Ekaterina Shelest, Johnny Debebe, Fei Sang, Marina Escalera Zamudio, Sarah Francois, Bernardo Gutierrez, Tetyana I. Vasylyeva, Flavia Flaviani, Manon Ragonnet-Cronin, Katherine L. Smollett, Alice Broos, Daniel Mair, Jenna Nichols, Kyriaki Nomikou, Lily Tong, Ioulia Tsatsani, Sarah O’Brien, Steven Rushton, Roy Sanderson, Jon Perkins, Seb Cotton, Abbie Gallagher, Elias Allara, Clare Pearson, David Bibby, Gavin Dabrera, Nicholas Ellaby, Eileen Gallagher, Jonathan Hubb, Angie Lackenby, David Lee, Nikos Manesis, Tamyo Mbisa, Steven Platt, Katherine A. Twohig, Mari Morgan, Alp Aydin, David J. Baker, Ebenezer Foster-Nyarko, Sophie J. Prosolek, Steven Rudder, Chris Baxter, Sílvia F. Carvalho, Deborah Lavin, Arun Mariappan, Clara Radulescu, Aditi Singh, Miao Tang, Helen Morcrette, Nadua Bayzid, Marius Cotic, Carlos E. Balcazar, Michael D. Gallagher, Daniel Maloney, Thomas D. Stanton, Kathleen A. Williamson, Robin Manley, Michelle L. Michelsen, Christine M. Sambles, David J. Studholme, Joanna Warwick-Dugdale, Richard Eccles, Matthew Gemmell, Richard Gregory, Margaret Hughes, Charlotte Nelson, Lucille Rainbow, Edith E. Vamos, Hermione J. Webster, Mark Whitehead, Claudia Wierzbicki, Adrienn Angyal, Luke R. Green, Max Whiteley, Iraad F. Bronner, Ben W. Farr, Stefanie V. Lensing, Shane A. McCarthy, Michael A. Quail, Nicholas M. Redshaw, Scott A. J. Thurston, Will Rowe, Amy Gaskin, Thanh Le-Viet, Jennifier Liddle, Ewan Birney, Erik Volz, Sebastian Funk, Dominic Kwiatkowski, Meera Chand, Inigo Martincorena, Jeffrey C. Barrett, Moritz Gerstung

**Affiliations:** 1 European Molecular Biology Laboratory, European Bioinformatics Institute EMBL-EBI, Hinxton, UK; 2 Wellcome Sanger Institute, Hinxton, UK; 3 The Francis Crick Institute, London, UK; 4 Public Health England, London, UK; 5 London School of Hygiene & Tropical Medicine, London, UK; 6 The Big Data Institute, Nuffield Department of Medicine, University of Oxford, Oxford, UK; 7 MRC Centre for Global Infectious Disease Analysis, Jameel Institute for Disease and Emergency Analytics, Imperial College London, London, UK; 8 Guy’s and St Thomas’ NHS Foundation Trust, London, UK; 9 Division for AI in Oncology, German Cancer Research Centre DKFZ, Heidelberg, Germany; 10 Department of Medicine, University of Cambridge, Cambridge, UK; 11 Centre for Enzyme Innovation, University of Portsmouth, Portsmouth, UK; 12 School of Pharmacy & Biomedical Sciences, University of Portsmouth, Portsmouth, UK; 13 Cardiff University, Cardiff, UK; 14 Public Health Wales, Cardiff, UK; 15 Institute of Microbiology and Infection, University of Birmingham, Birmingham, UK; 16 King’s College London, London, UK; 17 University of Edinburgh, Edinburgh, UK; 18 Centre for Clinical Infection and Diagnostics Research, Department of Infectious Diseases, Guy’s and St Thomas’ NHS Foundation Trust, London, UK; 19 University College London Hospitals NHS Foundation Trust, London, UK; 20 Advanced Pathogen Diagnostics Unit, University College London Hospital, London, UK; 21 Great Ormond Street Institute of Child Health, University College London, London, UK; 22 Department of Infectious Diseases and Microbiology, Cambridge University Hospitals NHS Foundation Trust, Cambridge, UK; 23 Division of Virology, Department of Pathology, University of Cambridge, Cambridge, UK; 24 University Hospital Southampton NHS Foundation Trust, Southampton, UK; 25 School of Health Sciences, University of Southampton, Southampton, UK; 26 School of Medicine, University of Southampton, Southampton, UK; 27 Division of Infection and Immunity, University College London, London, UK; 28 University of Brighton, Brighton, UK; 29 Institute for Infection and Immunity, St George’s University of London, London, UK; 30 Infection Care Group, St George’s University Hospitals NHS Foundation Trust, London, UK; 31 MRC–University of Glasgow Centre for Virus Research, Glasgow, UK; 32 University of Cambridge, Cambridge, UK; 33 Queen’s University Belfast, Belfast, UK; 34 Blackpool Teaching Hospitals NHS Foundation Trust, Blackpool, UK; 35 Hull University Teaching Hospitals NHS Trust, Hull, UK; 36 University Hospitals Dorset NHS Foundation Trust, Poole, UK; 37 University Hospitals Sussex NHS Foundation Trust, Worthing, UK; 38 University of Exeter, Exeter, UK; 39 Belfast Health & Social Care Trust, Belfast, UK; 40 Deep Seq, School of Life Sciences, Queens Medical Centre, University of Nottingham, Nottingham, UK; 41 East Kent Hospitals University NHS Foundation Trust, Canterbury, UK; 42 University of Kent, Canterbury, UK; 43 Hub for Biotechnology in the Built Environment, Northumbria University, Newcastle upon Tyne, UK; 44 Northumbria University, Newcastle upon Tyne, UK; 45 NU-OMICS, Northumbria University, Newcastle upon Tyne, UK; 46 Centre for Genomic Pathogen Surveillance, University of Oxford, Oxford, UK; 47 Public Health England, Cambridge, Cambridge, UK; 48 University of Sheffield, Sheffield, UK; 49 Portsmouth Hospitals University NHS Trust, Portsmouth, UK; 50 NHS Lothian, Edinburgh, UK; 51 NHS Greater Glasgow and Clyde, Glasgow, UK; 52 Quadram Institute Bioscience, Norwich, UK; 53 University of East Anglia, Norwich, UK; 54 University of Oxford, Oxford, UK; 55 Hampshire Hospitals NHS Foundation Trust, Basingstoke, UK; 56 Royal Free London NHS Foundation Trust, London, UK; 57 South Tees Hospitals NHS Foundation Trust, Middlesbrough, UK; 58 School of Biological Sciences, University of Portsmouth, Portsmouth, UK; 59 Public Health Scotland, Edinburgh, UK; 60 Health Services Laboratories, London, UK; 61 Heartlands Hospital, Birmingham, UK; 62 University of Liverpool, Liverpool, UK; 63 Department of Zoology, University of Oxford, Oxford, UK; 64 MRC Biostatistics Unit, University of Cambridge, Cambridge, UK; 65 Microbiology Department, Buckinghamshire Healthcare NHS Trust, Aylesbury, UK; 66 The Newcastle upon Tyne Hospitals NHS Foundation Trust, Newcastle upon Tyne, UK; 67 University of St Andrews, St Andrews, UK; 68 Imperial College Healthcare NHS Trust, London, UK; 69 NIHR Health Protection Research Unit in HCAI and AMR, Imperial College London, London, UK; 70 Cambridge University Hospitals NHS Foundation Trust, Cambridge, UK; 71 Department of Microbiology, South West London Pathology, London, UK; 72 Betsi Cadwaladr University Health Board, Wrexham, UK; 73 Institute of Biodiversity, Animal Health & Comparative Medicine, Glasgow, UK; 74 Norfolk County Council, Norfolk, UK; 75 Great Ormond Street Hospital for Children NHS Foundation Trust, London, UK; 76 Wellcome Centre for Human Genetics, Nuffield Department of Medicine, University of Oxford, Oxford, UK; 77 Cardiff and Vale University Health Board, Cardiff, UK; 78 Turnkey Laboratory, University of Birmingham, Birmingham, UK; 79 Norfolk and Norwich University Hospitals NHS Foundation Trust, Norwich, UK; 80 Royal Brompton and Harefield Hospitals, London, UK; 81 The Queen Elizabeth Hospital King’s Lynn NHS Foundation Trust, King’s Lynn, UK; 82 Whittington Health NHS Trust, London, UK; 83 Barking, Havering and Redbridge University Hospitals NHS Trust, London, UK; 84 Bournemouth University, Bournemouth, UK; 85 Clinical Microbiology Department, Queens Medical Centre, Nottingham University Hospitals NHS Trust, Nottingham, UK; 86 Clinical Microbiology, University Hospitals of Leicester NHS Trust, Leicester, UK; 87 County Durham and Darlington NHS Foundation Trust, Durham, UK; 88 Department of Microbiology, Kettering General Hospital, Kettering, UK; 89 Barts Health NHS Trust, London, UK; 90 North West London Pathology, London, UK; 91 Maidstone and Tunbridge Wells NHS Trust, Maidstone, UK; 92 Microbiology, Royal Oldham Hospital, Oldham, UK; 93 North Cumbria Integrated Care NHS Foundation Trust, Carlisle, UK; 94 North Tees and Hartlepool NHS Foundation Trust, Stockton on Tees, UK; 95 Path Links, Northern Lincolnshire and Goole NHS Foundation Trust, Grimsby, UK; 96 Queen Elizabeth Hospital, Birmingham, UK; 97 Royal Devon and Exeter NHS Foundation Trust, Exeter, UK; 98 Virology, School of Life Sciences, Queens Medical Centre, University of Nottingham, Nottingham, UK; 99 Swansea University, Swansea, UK; 100 Viapath, Guy’s and St Thomas’ NHS Foundation Trust, and King’s College Hospital NHS Foundation Trust, London, UK; 101 Sheffield Teaching Hospitals NHS Foundation Trust, Sheffield, UK; 102 Cambridge Stem Cell Institute, University of Cambridge, Cambridge, UK; 103 West of Scotland Specialist Virology Centre, NHS Greater Glasgow and Clyde, Glasgow, UK; 104 Public Health Agency, Belfast, UK; 105 Northumbria Healthcare NHS Foundation Trust, Newcastle upon Tyne, UK; 106 University of Southampton, Southampton, UK; 107 East Suffolk and North Essex NHS Foundation Trust, Colchester, UK; 108 East Sussex Healthcare NHS Trust, St Leonards-on-Sea, UK; 109 Gateshead Health NHS Foundation Trust, Gateshead, UK; 110 Isle of Wight NHS Trust, Newport, UK; 111 King’s College Hospital NHS Foundation Trust, London, UK; 112 Liverpool Clinical Laboratories, Liverpool, UK; 113 Manchester University NHS Foundation Trust, Manchester, UK; 114 North Middlesex University Hospital NHS Trust, London, UK; 115 Southwest Pathology Services, Taunton, UK; 116 The Royal Marsden NHS Foundation Trust, London, UK; 117 The Royal Wolverhampton NHS Trust, Wolverhampton, UK; 118 University of Birmingham, Birmingham, UK; 119 Watford General Hospital, Watford, UK; 120 Guy’s and St Thomas’ Biomedical Research Centre, London, UK; 121 Newcastle University, Newcastle, UK

**Keywords:** Evolutionary genetics, Viral infection, Epidemiology, SARS-CoV-2

Correction to: *Nature* 10.1038/s41586-021-04069-y Published online 14 October 2021

In the version of this article initially published, the name of author Erik Volz appeared as “Erik M. Volz” in The COVID-19 Genomics UK (COG-UK) Consortium contributions listings. Further, the affiliation listed for COG-UK Consortium member Adam A. Witney was shown incorrectly (Imperial College London) and has been now amended to the Institute for Infection and Immunity, St George’s Hospital of London, London, UK. The changes have been made to the HTML and PDF versions of the article.

